# Case Report: Successful immune checkpoint inhibitor rechallenge after sintilimab-induced Guillain-Barré syndrome

**DOI:** 10.3389/fimmu.2025.1546886

**Published:** 2025-03-19

**Authors:** Lin Ye, Wan Rong Yue, Hao Shi, Jian Ren Li, Yu Ya Qun

**Affiliations:** ^1^ Department of Hepatobiliary and Pancreatic Surgery, Affiliated Hospital of Guilin Medical University, Guilin, China; ^2^ Department of Pathology, Guilin People's Hospital, Guilin, China

**Keywords:** hepatocellular carcinoma, immune checkpoint inhibitor, immune-related adverse events, Guillain-Barré syndrome, neurologic adverse events, ICI rechallenge

## Abstract

Immune checkpoint inhibitors (ICIs) have revolutionized hepatocellular carcinoma (HCC) treatment, while immune-related adverse events (IRAEs) pose significant challenges. We report a 60-year-old male with unresectable HCC who developed Guillain-Barré syndrome (GBS), a rare but severe neurologic complication, after three cycles of sintilimab plus bevacizumab biosimilar and conventional transarterial chemoembolization (c-TACE). The patient presented with progressive ascending weakness, reaching symmetric quadriparesis with proximal muscle strength of 2/5 in upper limbs and 1/5 in lower limbs. Following sintilimab discontinuation, treatment with intravenous immunoglobulin (2 g/kg) and oral prednisone (30 mg/day) achieved complete neurological recovery within one month. Given the patient’s favorable initial tumor response and strong request, immunotherapy was cautiously reinstated using tislelizumab after thorough clinical evaluation. Following four cycles of treatment, significant tumor response enabled successful conversion surgery with major pathological response (necrosis rate >70%). With 26-month survival and no evidence of recurrence, this case demonstrates the potential feasibility of ICI rechallenge with an alternative PD-1 inhibitor following sintilimab-induced GBS. Our experience suggests that ICI-related neurological adverse events may be drug-specific rather than class-specific, potentially providing valuable treatment options for patients showing favorable tumor response despite experiencing severe IRAEs, though larger studies are needed for validation.

## 1 Introduction

Hepatocellular carcinoma (HCC) ranks as the fourth most common malignancy and represents the second leading cause of cancer-related mortality in China (1). In 2022, China reported 368,000 novel HCC cases, representing 42.4% of the global incidence, with 317,000 associated fatalities, accounting for 41.7% of worldwide mortality in this malignancy (2). Despite a substantial improvement in overall cancer survival rates from 30.9% to 40.5% between 2003-2015, HCC patients experienced only a marginal increase from 10.1% to 12.1% (3). Benefiting from comprehensive screening strategies, approximately 70% of patients in Japan and Taiwan are diagnosed at an early stage, with a high proportion meeting curative resection criteria. In contrast, 64% of patients in China are diagnosed at Barcelona Clinic Liver Cancer (BCLC) stages B or C (4). Curative surgical resection remains the gold standard for HCC treatment, with a 5-year survival rate of up to 64% (5). Consequently, conversion therapy, which reduces tumor size, downstaging, and creates conditions for subsequent surgery, is crucial to the patient’s long-term survival (6, 7).

With the ongoing advancement of interventional treatment techniques such as transcatheter arterial chemoembolization (TACE) and hepatic arterial infusion chemotherapy (HAIC), along with the emergence of novel targeted drugs and immune checkpoint inhibitors (ICIs), multiple conversion therapy options have become available for intermediate and advanced HCC (8). Numerous studies have shown that monotherapy generally offers limited efficacy and seldom results in long-term benefits. The integration of multiple therapeutic modalities combined with personalized treatment protocols represents the current predominant trend in medical practice (9).

As ICIs gain widespread adoption in clinical practice, the recognition and management of (immune-related adverse events) IRAEs have become critical clinical challenges. These events can impact any organ system, predominantly due to enhanced T cell activation, autoimmune responses, and dysregulated inflammatory reactions (10). Although most IRAEs are mild and manageable, certain severe complications pose life-threatening risks, requiring immediate intervention and standardized management.

Guillain-Barré syndrome (GBS) represents an exceedingly rare, yet severe neurologic IRAEs characterized by progressive ascending paralysis. Clinical manifestations typically include symmetric muscle weakness, loss of deep tendon reflexes, and sensory abnormalities (11). The pathogenesis of ICI-induced GBS remains incompletely understood but likely involves multiple mechanisms, including loss of peripheral immune tolerance, regulatory T cell dysfunction, and molecular mimicry between tumor antigens and neural tissues (12).

Current guidelines advocate for the permanent discontinuation of ICIs upon the occurrence of severe neurologic adverse events (NAEs). Nevertheless, this presents a therapeutic conundrum for patients who have demonstrated substantial antitumor responses. In contexts with limited therapeutic alternatives, the potential for a rechallenge with alternate ICIs has not been adequately investigated (13). This report describes a case of successful tislelizumab rechallenge in a patient with unresectable HCC following sintilimab-induced GBS, providing valuable clinical insights into the management of patients who demonstrate therapeutic response to immunotherapy despite experiencing NAEs.

## 2 Case presentation

A 60-year-old male patient, with a height of 172 cm and weight of 65 kg, had been managing chronic hepatitis B for 20 years and was consistently taking Tenofovir (Hepsera^®^) 300 mg daily for antiviral therapy. A large space-occupying lesion in the right hepatic lobe was incidentally discovered during a routine medical screening examination. The patient presented with an (Eastern Cooperative Oncology Group) ECOG performance status of 1 and Child-Pugh class B liver function, Laboratory tests revealed elevated (serum alpha-fetoprotein) AFP (2,356 ng/mL) and (protein induced by vitamin K absence or antagonist-II)PIVKA-II (35,680 mAU/mL) levels, with HBV-DNA less than 100 IU/mL. Integration of laboratory and imaging findings established the diagnosis of HCC, BCLC stage C. Our multidisciplinary team (MDT) determined tumor unresectability based on three key factors: elevated (indocyanine green retention rate at 15 minutes) ICG-R15 (18.5%), insufficient remnant liver volume and inadequate surgical margins due to tumor proximity (5 mm) to the middle hepatic vein. Therefore, a combination therapy regimen was established, consisting of conventional transarterial chemoembolization (c-TACE) plus sintilimab (TYVYT^®^) and bevacizumab biosimilar (BYVASDA^®^).

Initially, the patient underwent c-TACE with a mixture of epirubicin (40 mg) and lipiodol (20 mL), followed by embolization with two vials of CalliSpheres^®^ blank microspheres (100-300 μm). Intraoperative angiography demonstrated a significant reduction in tumor vascularity with satisfactory lipiodol retention. The procedure was completed successfully without complications. Six days following c-TACE, Sintilimab immunotherapy was initiated with a standard three-week cycle regimen. One week after completing the third cycle of sintilimab, the patient developed progressive, symmetrical ascending weakness without sensory loss. Three weeks later, the weakness progressed to inability to stand and upper limb weakness, accompanied by mild dysphagia and dyspnea, but without ptosis, neck weakness, or respiratory compromise.

Upon emergency admission, physical examination revealed symmetric quadriparesis with proximal muscle strength of 2/5 in upper limbs and 1/5 in lower limbs, and distal strength of 3/5 and 2/5, respectively. Deep and superficial reflexes were absent bilaterally with negative Babinski signs and stocking-glove sensory deficit. Cerebrospinal fluid (CSF) analysis showed elevated pressure (145 mmH_2_O) and protein (128 mg/dL) with normal cell count (3×10^6^/L), glucose (3.6 mmol/L), and chloride (120 mmol/L), demonstrating albuminocytologic dissociation. Nerve conduction studies revealed reduced conduction velocities in motor nerves (median: 32 m/s; common peroneal: 28 m/s) and sensory nerves (median: 35 m/s; sural: 30 m/s), with prolonged F-wave latencies (median nerve: 35 ms; peroneal nerve: 38 ms), indicating peripheral neuropathy.

The patient was diagnosed with sintilimab-induced GBS. Sintilimab was discontinued, and treatment was initiated with intravenous immunoglobulin (IVIG) at 2 g/kg (total 130 g divided into 26 g daily for 5 days) and oral prednisone (30 mg/day, divided into 15 mg in the morning and afternoon). The treatment regimen also included twice-daily bedside rehabilitation (30 minutes per session), high-protein diet supplementation with B vitamins, and prophylactic subcutaneous enoxaparin (4,000 U/day). The patient showed gradual improvement. After one week of GBS treatment, muscle strength improved to 3/5 proximally and 4/5 distally in the upper extremities, and 2/5 proximally and 3/5 distally in the lower extremities, with significant improvement in swallowing function. At discharge (day 14), muscle strength reached 4/5 in all extremities, enabling assisted standing with a walker, and swallowing function had largely recovered. Discharge instructions included continued prednisone (20 mg/day) with dose adjustment after two weeks, and ongoing rehabilitation.

**Figure 1:**
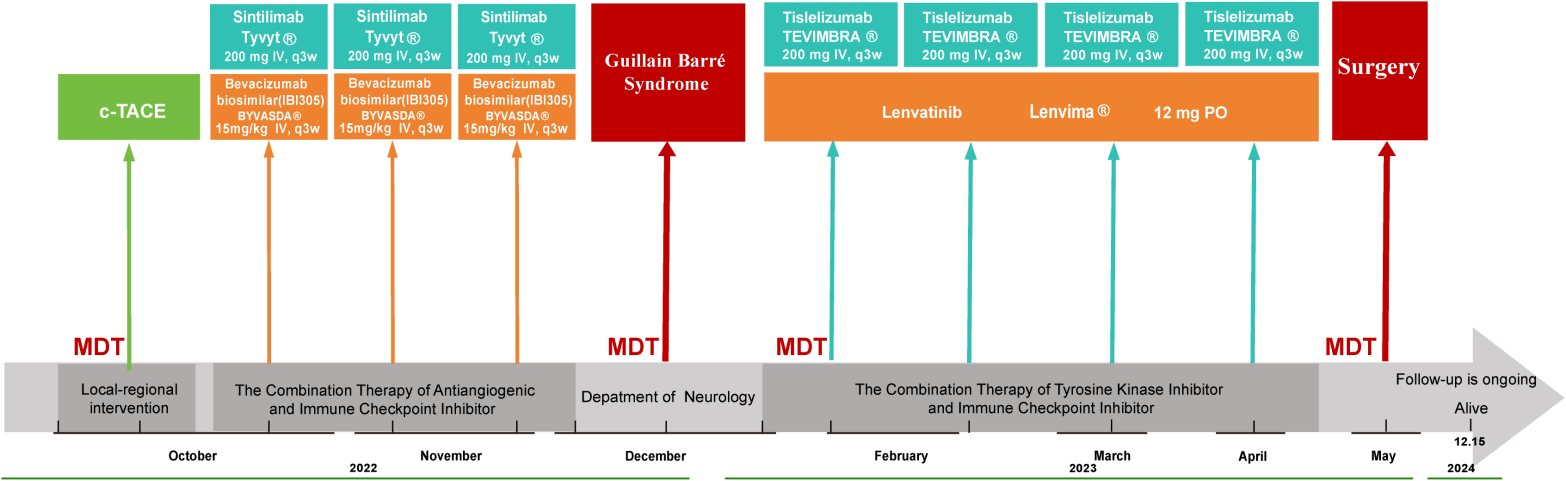
Schematic illustration of the treatment timeline. c-TACE Conventional Transarterial Chemoembolization, MDT Multidisciplinary Team, IV Intravenous.

**Figure 2:**
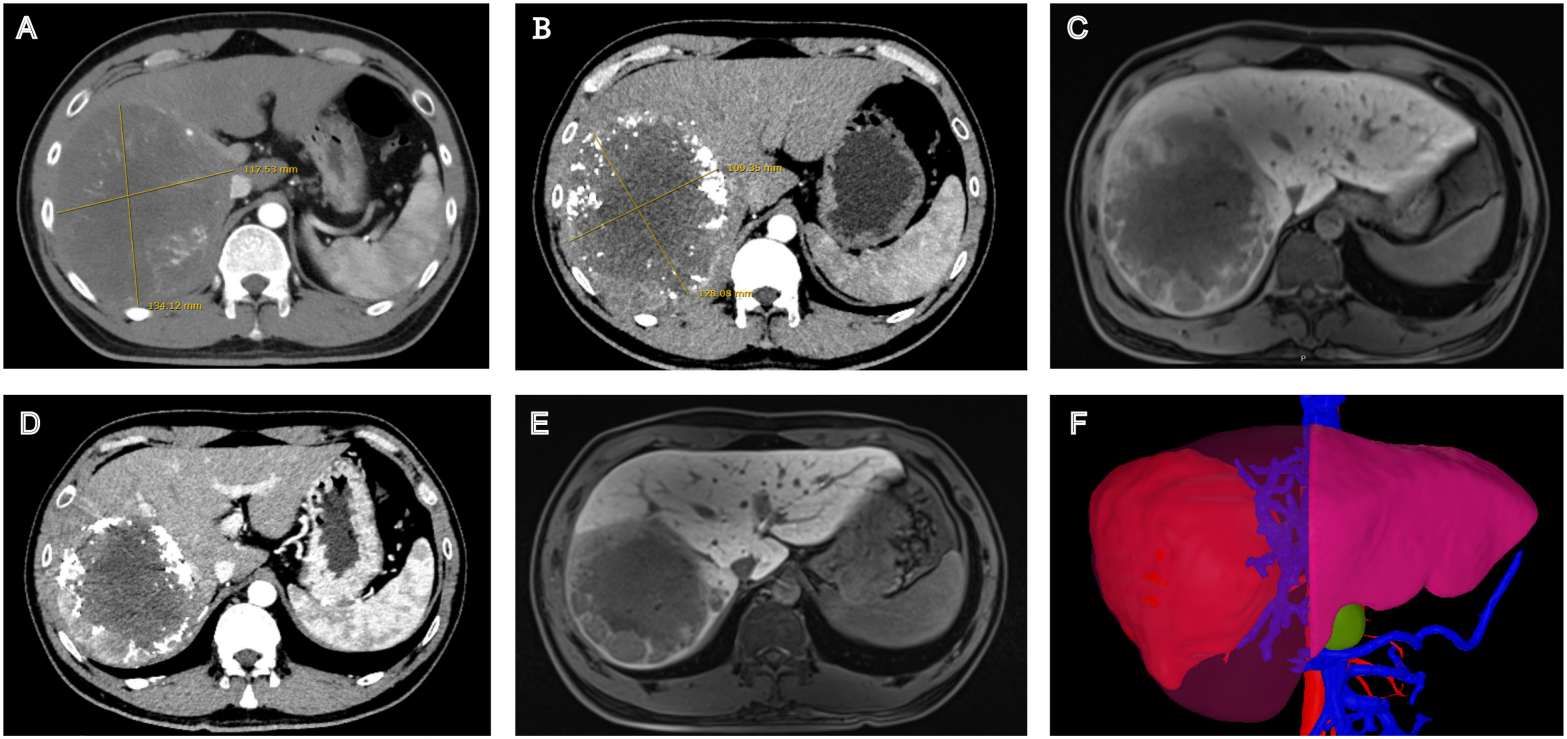
Radiological changes during the patient’s treatment course. **(A)** Contrast-enhanced CT at baseline. **(B)** Contrast-enhanced CT After c-TACE therapy. **(C)** Gd-EOB-DTPA-enhanced MR after c-TACE therapy. **(D)** Contrast-enhanced CT after sintilimab (3 cycles) and Tislelizumab (4 cycles). **(E)** Gd-EOB-DTPA-enhanced MR after sintilimab (3 cycles) and Tislelizumab (4 cycles). **(F)** Three-dimensional volumetric analysis based on contrast-enhanced CT before surgery.

Following complete neurological recovery and approximately two months after the onset of GBS, immunotherapy was reinstated upon the patient’s strong request and thorough clinical evaluation, Sintilimab was substituted with tislelizumab, which was administered for four cycles over a two-month period. Following the standard treatment course, imaging evaluation demonstrated significant tumor response, enabling surgical intervention. Pathological examination revealed a major pathological response (MPR) with tumor necrosis rate >70%. The complete treatment course is illustrated in **Figure 1**, and radiological changes are shown in **Figure 2**. As of December 15, 2024, the patient remains alive with an overall survival (OS) of 26 months. Serum tumor markers showed a sustained decrease. No evidence of recurrence or metastasis was observed during follow-up.

## 3 Discussion

Due to the heterogeneity of HCC and divergent treatment paradigms between Eastern and Western regions, official treatment guidelines often differ (14). For instance, the Chinese guidelines recommend TACE, hepatic resection, and radiotherapy for selected advanced HCC patients, while these options are not endorsed in Western guidelines. Mounting clinical evidence demonstrates that locoregional therapies achieve significantly higher local control rates for intrahepatic lesions compared to systemic treatment alone. In Chinese clinical practice, combination strategies incorporating both locoregional and systemic therapies are predominantly employed, while monotherapy with either tyrosine kinase inhibitors (TKIs) or ICIs is rarely utilized (15).

For unresectable hepatocellular carcinoma (uHCC), TACE is widely accepted as a first-line treatment modality. This approach is also recommended for advanced HCC patients with portal vein tumor thrombosis (PVTT) in Korea, Japan, and other Asian countries (16, 17). TACE combined with molecular targeted therapy is based on TKIs’ ability to suppress hypoxia-induced angiogenesis post-TACE. This synergistic approach enhances therapeutic efficacy through multiple mechanisms. TACE-induced tumor necrosis releases tumor antigens, enhancing antitumor immune responses, creating a pro-vascular environment for targeted therapy, and augmenting CD8+ T cell responses to tumor-associated antigens (TAAs). Immune checkpoint inhibitors complement this effect by blocking CTLA-4 and PD-1 pathways (18). A multicenter prospective randomized study in Japan and Korea reported complete or partial response rates of 73% and 2-year OS rates of 75% with TACE treatment (19). The CHANCE001 study demonstrated that TACE combined with targeted immunotherapy significantly improved outcomes compared to TACE alone (20). In contrast to previous studies incorporating various ICIs and targeted agents, the CHANCE2211 trial streamlined the therapeutic approach by evaluating the combination of carrelizumab and apatinib with TACE. Results demonstrated that this combination therapy significantly improved OS, progression-free survival (PFS), and objective response rate (ORR) compared to TACE monotherapy (21).

Recent advances in systemic therapy have not only created opportunities for surgical resection in initially unresectable patients but also effectively reduced postoperative recurrence and metastasis rates, thereby improving long-term survival benefits. The global phase III IMbrave150 trial demonstrated that atezolizumab plus bevacizumab significantly prolonged median OS and PFS compared to sorafenib, reducing the risk of death by 34% and disease progression by 35%. In the Chinese subgroup analysis, the combination therapy showed even more pronounced clinical benefits, with a 47% reduction in mortality risk and a 40% reduction in disease progression risk compared to sorafenib (22). The ORIENT-32 trial demonstrated that sintilimab plus bevacizumab biosimilar significantly outperformed sorafenib, with a 43% reduction in mortality risk and a 44% reduction in disease progression risk. This combination therapy has been approved in China as a first-line treatment for patients with unresectable or metastatic HCC who have not received prior systemic therapy (23). The BGB-A317-211 trial evaluated tislelizumab plus lenvatinib as first-line therapy for advanced HCC. The combination demonstrated statistical superiority in ORR compared to historical data on lenvatinib monotherapy. Notably, tumor shrinkage was observed in over 70% of patients, representing significant clinical implications from a surgical perspective (24). The global phase III CARES-310 trial, evaluating Camrelizumab plus apatinib as first-line therapy for advanced uHCC, achieved the longest median overall survival (25). Additionally, the HIMALAYA, which first reported 5-year overall survival data, led to regulatory approval from the Food and Drug Administration(FDA), European Medicines Agency(EMA), and Pharmaceuticals and Medical Devices Agency(PMDA) for the STRIDE regimen (tremelimumab plus durvalumab) in the treatment of uHCC (26).

The therapeutic landscape of HCC has entered the immunotherapy era, marked by the broad implementation of ICIs across diverse clinical settings, encompassing palliative, adjuvant, neoadjuvant, and multimodal treatment strategies (27). While providing clinical benefits, ICIs can disrupt immune homeostasis, resulting in IRAEs with an incidence rate of 66-72%. These events commonly affect target organs including skin, gastrointestinal tract, lungs, liver, and endocrine systems. Although NAEs are relatively rare, accounting for 1.0-12.0% of all reported IRAEs, complications such as encephalitis, GBS, and myasthenia gravis can potentially progress to severe or fatal outcomes (28–30). The Common Terminology Criteria for Adverse Events (CTCAE) provides a standardized grading system for irAE severity. Moderate (grade 2) to severe (grades 3-4) IRAEs can result in substantial organ dysfunction, deterioration of quality of life, and potential mortality, underscoring the critical importance of early recognition and appropriate therapeutic intervention (31, 32).

ICI-associated NAEs can manifest in various forms, including myasthenia gravis, facial nerve palsy, meningitis, hypophysitis, meningoradiculoneuritis, cerebellitis, transverse myelitis, and GBS (33). GBS is a particularly concerning neurological complication that can progress to life-threatening muscle weakness and autonomic dysfunction. While classical GBS is typically triggered by known or unknown immune stimuli such as infections, surgery, vaccination, or trauma, certain medications, including ICIs, can also precipitate this condition (11). Electrophysiological findings suggest that immune-related GBS subtypes primarily present as widespread sensorimotor polyneuropathy characterized by acute inflammatory demyelinating polyneuropathy (AIDP) or acute motor axonal neuropathy (AMAN). Overall, the clinical course, severity, and outcomes of GBS demonstrate substantial heterogeneity (34).

The exact pathogenic mechanism of ICI-induced GBS remains unclear, studies suggest that ICIs promote T-cell proliferation and activation, leading to immune-mediated neuronal injury and subsequent autoimmune neuropathy. Research has demonstrated that both human and murine neurons express PD-L1 molecules, with significantly increased expression during neuronal injury. Moreover, neurons, like tumor cells, can express antigens recognizable by T cells. During ICI therapy, particularly with dual ICI combinations (anti-CTLA-4 and anti-PD-1 antibodies), effector T cells may directly target neurons, triggering autoimmune neurological disorders. Currently, available CTLA-4-targeted ICIs, such as the humanized monoclonal antibody ipilimumab, block the CTLA-4-B7 pathway, enhancing effector T-cell proliferation and activation, thereby augmenting antitumor immune responses and tumor cell destruction (35, 36). The potential mechanisms of IRAEs induced by anti-PD-1 antibodies (nivolumab and pembrolizumab) or anti-PD-L1 antibodies (atezolizumab) are associated with PD-1/PD-L1 pathway inhibition, leading to excessive T-cell activation and reduced regulatory T-cell function. This results in enhanced toxicity of macrophages and neutrophils, release of interferon-gamma and tumor necrosis factor, and antibody production by B cells (37, 38).

The literature comparing the propensity of different PD-1 inhibitors to induce IRAEs remains limited. However, emerging evidence suggests that structural and pharmacological differences between PD-1 inhibitors may influence their safety profiles. Sintilimab, tislelizumab, and other PD-1 inhibitors differ in their binding epitopes and affinities, which could explain variations in their immunological effects and adverse event profiles. Large-scale clinical trial data demonstrates that in the RATIONALE-301 study, tislelizumab showed a grade 3-4 IRAE incidence rate of 18.2%, while sintilimab in the ORIENT-32 study had a corresponding rate of 24.4% (23, 39). Specifically regarding neurological adverse events, the CheckMate-459 study showed that nivolumab led to a 2.3% incidence of neurological IRAEs, while pembrolizumab in the KEYNOTE-240 study had an incidence rate of 1.8% (40, 41). In this case, the patient did not experience NAEs after switching to tislelizumab. This may be related to differences in immune system activation patterns between the two drugs or individual patient variations. Tislelizumab’s binding region differs slightly from other PD-1 inhibitors, potentially leading to unique characteristics in its immune system activation (42).

The literature on ICIs-induced GBS and experiences of ICI rechallenge is limited, as only a small number of cases have been reported, summarized in **Table 1**. In the case of sintilimab, research has not revealed any unexpected side effects or off-target reactions, with most adverse events being grade 1-2 and not requiring specialized treatment, apart from thyroid dysfunction, colitis, and hepatitis, most complications are rare, Notable reported cases of these rare IRAEs include ICI-associated myocarditis, toxic epidermal necrolysis (TEN), severe erosive hemorrhagic gastritis, and pyloric obstruction (43–45).

**Table 1:** Case reports/series of checkpoint inhibitor-induced GBS.

Cases	Diseases	Checkpoint inhibitors	IRAEs	Treatment	Outcome^#^	Time(Months)^*^	Rechallenge	DOI
Mengge Ding et al.	NSCLC	KN046	GBS	MMF	Recovery	>8	NO	10.3389/fimmu.2023.1132692
Tomoyo Oguri et al.	Lung adenocarcinoma	Pembrolizumab	GBS+SJS/TEN	IVIG	Death	>2	NO	10.1177/232470962110374
Nicholas Gravbrot et al.	Melanoma	Ipilimumab	GBS	IVIG Prednisone	Recovery	>24	YES(Pembrolizumab)	10.1155/2019/5490707
Jialing Li et al.	Lung adenocarcinoma	Tislelizumab	GBS	IVIG Acupuncture	Recovery	>12	Not mentioned	10.3389/fneur.2022.908282
Kensuke Okada et al.	Renal cell cancer	Nivolumab	GBS(CIDP)	Prednisolone, IVMP Corticosteroids	Recovery	>24	NO	10.1007/s00415-020-10213-x
	Gastric cancer	Nivolumab	GBS(CIDP)	IVIG, IVMP Prednisolone	Recovery	>9	NO	
	Gastric cancer	Nivolumab	GBS(CIDP)	IVMP	Recovery	Not mentioned	Not mentioned	
	NSCLC	Atezolizumab	GBS(CIDP)	IVIG, IVMP Prednisolone	Recovery	Not mentioned	Not mentioned	
Heesung Moon et al.	Ovarian cancer	Pembrolizumab	Polyneuropathy	IVIG Methylprednisolone	Recovery	>10	NO	10.1097/MD.0000000000030236
Desheng Zhang et al.	Gastric cancer	Toripalimab	GBS	IVIG Methylprednisolone Rituximab	Recovery	>8	NO	10.3389/fneur.2024.1348304
Tal Sharon et al.	Pharyngeal SCC	Nivolumab	GBS	IVIG, IVMP Prednisolone	Not mentioned	Not mentioned	NO	10.7759/cureus.69575
Vikram Sangani et al.	Bladder cancer	Pembrolizumab	GBS	IVIG	Recovery	Not mentioned	NO	10.1080/20009666.2021.1903133

^#^Whether Guillain-Barré syndrome resulted in clinical recovery or fatal outcome.

^*^Time from onset of Guillain-Barré syndrome to death from any cause.

IRAEs immune-related adverse events, NSCLC non-small cell lung cancer, KN046 bispecific antibody targeting PD-L1 and CTLA-4, GBS Guillain-Barré syndrome, SJS/TEN Stevens-Johnson syndrome/toxic epidermal necrosis, IVIG intravenous immunoglobulin, IVMP intravenous methylprednisolone, CIDP chronic inflammatory demyelinating polyradiculoneuropathy, AIDP acute inflammatory demyelinating polyneuropathy, SCC Squamous Cell Carcinoma.

To our knowledge, this report describes the first case of a successful transition from sintilimab-related GBS to tislelizumab, with a successful immune rechallenge leading to conversion surgery. Treatment decisions, in this case, were facilitated by the patient’s high educational background and favorable socioeconomic circumstances, with therapeutic strategies determined through a shared decision-making process incorporating both multidisciplinary team (MDT) recommendations and patient preferences factors that merit careful consideration in clinical practice. The diagnosis of GBS in this case was established based on clinical history and examination, supported by ancillary investigations including cerebrospinal fluid analysis and electrophysiological studies. Upon high clinical suspicion, ICI therapy was immediately discontinued, and treatment with immunoglobulin and corticosteroids was initiated. According to the American Society of Clinical Oncology (ASCO) Clinical Practice Guidelines and National Comprehensive Cancer Network (NCCN) Guidelines, Corticosteroids and IVIG represent conventional therapeutic approaches for ICI-associated GBS. This combination therapy has demonstrated clinical improvement in 73% of patients (46, 47). Methylprednisolone at 1-2 mg/kg may be considered, particularly in GBS patients with cerebrospinal fluid pleocytosis exceeding expected levels (48). Plasma exchange (PE) may be employed as second-line therapy when initial treatments prove ineffective. However, its efficacy as a first-line treatment remains unclear (49). Physical therapy (PT) constitutes an essential component in the rehabilitation and management of GBS. In our case, the patient underwent one month of physical therapy, achieving complete neurological recovery (50).

Another critical consideration in clinical practice is the safety of resuming ICI therapy after adverse event resolution. Due to limited case numbers, literature specifically addressing cancer-specific therapy reinitiation following checkpoint inhibitor-induced GBS is scarce. Most protocols recommend discontinuation of ICI therapy in cases of severe adverse events. The NCCN guidelines further specify that severe irAEs from one class of immunotherapy necessitate permanent discontinuation of the same class, with moderate irAEs warranting cautious consideration. A retrospective study in melanoma patients suggests that toxicity may be treatment-specific rather than universal across different types of immune checkpoint blockade (51). A study investigating tremelimumab/durvalumab as an ICI rechallenge option after initial atezolizumab/bevacizumab therapy demonstrated satisfactory early safety and efficacy profiles (52). According to CTCAE criteria, this case was classified as grade 2 (53). Following discontinuation of sintilimab (anti-PD-1), the patient underwent a successful immune rechallenge with an alternative anti-PD-1 agent, tislelizumab. This conversion therapy ultimately transformed unresectable disease to surgically resectable status, achieving long-term survival benefits without recurrence of neurological symptoms.

This study offers novel therapeutic insights for managing NAEs associated with ICI therapy. For patients who initially respond well to immunotherapy, switching to an alternative PD-1 inhibitor may be a safe and viable option, even in cases of ICIs-induced GBS. This approach, implemented through systematic MDT discussions, provided timely and scientifically sound treatment decisions for patients with limited therapeutic alternatives.

## 4 Conclusion

ICIs have demonstrated significant efficacy in the treatment of HCC, but the management of IRAEs remains challenging. This study presents a rare but clinically significant case of a patient with sindilimab-induced GBS. After receiving standard treatment and fully recovering, cautiously selecting another PD-1 inhibitor for rechallenge was a potentially feasible treatment option, which at least brought long-term survival benefits to this patient. Moreover, this case successfully achieved a conversion from unresectable to radical resection, fully reflecting the value of individualized treatment strategies. Although the experience of a single case has certain limitations, it is a worthwhile attempt in the absence of standardized treatment regimens. Moving ahead, several crucial research directions need to be explored, including large-scale multicenter studies to validate the safety and efficacy of ICI rechallenge, investigations of differential IRAE profiles among PD-1 inhibitors, and development of predictive biomarkers for patient selection. Additionally, comprehensive assessment of patient-reported outcomes during rechallenge will be essential for clinical decision-making. We anticipate that future research in these areas will help establish evidence-based guidelines for ICI rechallenge in clinical practice.

## Data Availability

The original contributions presented in the study are included in the article/supplementary material. Further inquiries can be directed to the corresponding author.
